# Memory Deficits in Schizophrenia: A Selective Review of Functional Magnetic Resonance Imaging (fMRI) Studies

**DOI:** 10.3390/bs3030330

**Published:** 2013-06-27

**Authors:** Nina V. Kraguljac, Annusha Srivastava, Adrienne C. Lahti

**Affiliations:** Department of Psychiatry and Behavioral Neurobiology, University of Alabama at Birmingham, SC 501, 1530 3^rd^ Ave S, Birmingham, AL 35294, USA; E-Mails: nkraguljac@uab.edu (N.V.K.); annusha16@yahoo.com (A.S.)

**Keywords:** schizophrenia, memory, functional magnetic resonance imaging (fMRI), dorsolateral prefrontal cortex, hippocampus, medial temporal lobe

## Abstract

Schizophrenia is a complex chronic mental illness that is characterized by positive, negative and cognitive symptoms. Cognitive deficits are most predictive of long-term outcomes, with abnormalities in memory being the most robust finding. The advent of functional magnetic resonance imaging (fMRI) has allowed exploring neural correlates of memory deficits *in vivo.* In this article, we will give a selective review of fMRI studies probing brain regions and functional networks that are thought to be related to abnormal memory performance in two memory systems prominently affected in schizophrenia; working memory and episodic memory. We revisit the classic “hypofrontality” hypothesis of working memory deficits and explore evidence for frontotemporal dysconnectivity underlying episodic memory abnormalities. We conclude that fMRI studies of memory deficits in schizophrenia are far from universal. However, the current literature does suggest that alterations are not isolated to a few brain regions, but are characterized by abnormalities within large-scale brain networks.

## 1. Introduction

Schizophrenia is a chronic mental illness without racial or socioeconomic prejudice. It affects approximately one percent of the population worldwide and is a leading cause for disability and premature mortality in developed countries [1,2,3]. 

The disorder is characterized by three complexes of clinical features. Positive symptoms present as hallucinations, delusions and disorganization in thoughts or behaviors. Negative symptoms include lack of motivation, social withdrawal, diminished emotional expression/recognition of emotions and poverty of thinking and speech. Impaired cognition, including disturbance of memory, is present in approximately 75–85% of patients; patients typically score about one standard deviation lower on standardized assessments than would be expected for the general population [4,5,6]. 

Deficits are reported at illness onset and possibly even predate clinical manifestation; impairments are pervasive and may worsen with illness progression [7,8,9,10,11]. Cognitive dysfunction also is reported in high risk individuals and unaffected family members of patients with schizophrenia, suggesting significant genetic contribution [12,13,14]. If cognitive operations are disturbed, constructing something meaningful from incoming information may become inefficient [15]. Taken together, cognitive deficits may be one of the core abnormalities of schizophrenia that set the stage for later emergence of psychosis [16]. Not surprisingly, the extent of cognitive deficits is most predictive of long-term outcome and social functioning [17,18].

Cognitive deficits are most prominent in areas, such as working memory and episodic memory, attention, language comprehension, processing speed and executive function [19,20,21]; a meta-analysis reported the effect sizes for dysfunction to be largest in learning and memory [5]. Despite heterogeneity of the illness and the wide range of deficits, a number of findings appear to be consistent throughout the literature. Memory is the cognitive domain showing the most pronounced deficits; with working memory and episodic memory appearing to be primarily affected [22,23,24]. Importantly, the deficits are not just due to distractive positive symptoms, poor concentration or medication effects [25,26,27]. 

The underlying etiopathogenesis of memory deficits is believed to be complex and to widely affect neural circuitry. It appears that patients with schizophrenia may differ in the way in which they organize neural activity, maintain levels of activation in large-scale networks and functionally integrate neural circuits when compared to healthy controls. Abnormalities causing aberrant circuitry could be located (1) within a single region of the brain, (2) within a specific network or (3) at the level of interconnected networks [28,29]. While strictly localized pathophysiology may account for some aspects, memory deficits, abnormalities in interaction or integration of networks may hold a more sufficient explanation for the complexity of clinical presentation [30]. 

The advent of functional magnetic resonance imaging (fMRI) has provided a new opportunity to gain insight to neuronal activity and neural circuitry *in vivo* without requiring the subject to ingest substances, be exposed to radiation or undergo invasive procedures. Classic fMRI paradigms measure the change in blood oxygen level dependent (BOLD) signal evoked by specific tasks. The BOLD signal is a surrogate marker of neural activity; an increase in the signal is thought to reflect higher activation in the brain [31]. More recently, low frequency fluctuations of the BOLD signal during rest, *i.e.*, in absence of a specific task, have been employed to investigate temporal correlations across cortical areas that reflect functionally related brain networks [32,33].

To appreciate functional abnormalities that are present in patients with schizophrenia, one must first understand the general construct of memory. It is generally accepted that multiple independent, but interacting, memory systems exist [34,35]. These are conceptualized as organized structures of elementary operating components that consist of a neural substrate and its cognitive or behavioral correlates. While some components are shared by some or all systems, others are unique to specific memory systems [36]. Several steps in the process of memory formation have been identified: (1) encoding (information converted into a construct that is stored in the brain) (2) manipulation and maintenance of information and (3) retrieval (access of stored information). Neural substrates of both long- and short-term memory systems include the prefrontal cortex and medial temporal lobe (MTL), implicating a relationship between short-term memory and long-term memory [37]. 

While it is generally accepted that patients with schizophrenia often do poorly on memory tests, the exact nature of these deficits has a long history of being subject to debate [38,39]. Much of the empirical evidence comes from studies contrasting different memory systems in patients with schizophrenia to those of healthy controls [40], with an apparent pattern of working memory and episodic memory impairment, but less disruption of verbal short-term memory and procedural memory is seen in schizophrenia [41,42]. 

Working memory was originally defined as an active short-term process consisting of a central executive and a modality-specific slave system and can be conceptualized as a temporary store, whose contents are continually modified in response to immediate processing demands, but do not necessarily translate into long-term storage [43,44]. General consensus for the neuroanatomy of normal working memory includes the prefrontal cortex; however, it remains controversial if the hippocampal formation is contributing to working memory processes or if hippocampal activation found in some studies is because paradigms exceed the working memory span and start to involve long-term memory processes [45]. 

Episodic memory is a category of long-term memory that was originally defined as a record of a person’s experience that includes temporally dated information and spatio-temporal relations [46]. The neuroanatomy of episodic memory is less clearly delineated, but is thought to consist of a widely distributed network of cortical and subcortical brain regions and to be crucially dependent on the MTL, with contributions from the prefrontal cortex [47,48,49]. 

Importantly, these systems show similar activation patterns with tasks, suggesting that they share basic processing components [50,51,52]. Within the prefrontal cortex, the dorsolateral, ventrolateral and dorsal anterior cingulate cortices are recruited in both working memory and episodic memory tasks [50,53,54]. The anterior cingulate cortex supports dynamic adjustments in cognitive control, the ventrolateral prefrontal cortex is associated with item-specific information processing and the dorsolateral prefrontal cortex (DLPFC) is linked to processing of relations among items [55,56,57,58,59,60,61,62]. While evidence suggests that some basic memory processes are shared across systems, system-specific activation patterns are also present [53]. For example, working memory is associated with larger dorsolateral prefrontal cortex activation and episodic memory tasks elicit specific frontopolar activations [50,63,64]. When both memory systems are investigated in the same group of healthy controls, a common fronto-parieto-cerebellar network for working memory and episodic retrieval with common and specific activation of subregions in the prefrontal cortex and common activation of the MTL was identified [64]. 

Not surprisingly, these are the very areas that neuroimaging research of memory deficits in schizophrenia has most widely explored, but the nature of functional abnormalities during memory tasks in patients with schizophrenia remains controversial, because of inconsistencies in reports. The goal of this article is to give a selective review of fMRI studies that explore patterns of neural activation of memory processes in patients with schizophrenia. Evidence of neural abnormalities will be discussed in the context of two systems widely affected in schizophrenia; working memory and episodic memory [65,66].

## 2. Overview of fMRI Studies Relevant to Memory in Schizophrenia

### 2.1. Working Memory Deficits

The prefrontal cortex is one of the most widely explored regions in functional imaging studies of working memory. Reduced activation patterns in the DLPFC in schizophrenia in working memory tasks appear to be most widely replicated; with the extent of reduction being correlated with poorer performance [67,68]. For example, Callicott *et al*. report a failure of activation of the DLPFC in five of six patients with schizophrenia using a working memory task [69]. These abnormalities were later confirmed in groups of chronically ill and first episode patients, in medicated and medication naive subjects [70,71,72,73]. The failure of activity increases during task in medicated patients subsequently was reported only in high, but not in low, levels of working memory load. Not surprisingly, an association between decreased task performance and failure of activation was observed [74]. However, several studies are inconsistent with the finding of decreased prefrontal cortex activation during working memory tasks. No abnormalities in DLPFC activation patterns were found in acutely ill, medicated patients and hospitalized subjects in partial remission [75,76], and even hyperactivation was reported in mildly to moderately ill chronic patients with schizophrenia [77,78,79]. 

It has been suggested that the inconsistencies in the literature could be related to task requirements or may be explained by poor task performance in schizophrenia [28,80]. Many argue for the conceptualization of task related activation patterns as a non-linear, inverted U-shaped function that relates the fMRI signal to working memory load and is shifted to the left in patients with schizophrenia. Assuming an initial relative overactivation of this region with relatively low memory demands, a decline in processing capacity with increasing demand would then be accompanied by a relative underactivation [81,82,83]. A disruption in this activation-performance relationship in schizophrenia has indeed been reported [28,84]. Furthermore, a left shift of the curve was confirmed in a study of ten chronically medicated patients reaching peak activation on the working memory system at lower processing loads than healthy controls and a subsequent decline of DLPFC activity at high processing loads [85].

Honey *et al*. attempted to control for possible task performance confounds by recruiting 20 male patients with intact performance on a low-load verbal memory task and matched controls. In controls, functional response and behavioral performance correlated, but a de-coupling between these parameters was present in schizophrenia [86]. Accounting for performance differences on measures of cognition between patients with schizophrenia and healthy controls is a challenging problem. Controlling for these differences has been handled in different ways, including limiting analyses to correct trials or blocks of trials with acceptable accuracy, using task performance as a covariate, matching groups based on performance or adjusting disparity between task presentations to individual ability levels [75,83,87,88,89,90,91]. 

Interestingly, it appears that even patients who are able to keep up with processing demands tend to engage greater levels of activation or a less focused cortical activity state, thus responding to demands less efficiently [92,93]. Ragland *et al*. report that, while healthy participants respond to increased processing demands through an increase in DLPFC activity, patients with schizophrenia did not show higher DLPFC activity, but diffusely engaged a number of cortical and subcortical regions to meet the same demands [94]. In addition to reduced activation of the right DLPFC, an abnormal correlation between the left DLPFC and the left hippocampal formation that was not present in healthy controls was seen in a group of unmedicated patients, again underscoring widespread neural abnormalities [95]. In a quantitative meta-analysis of twelve studies using a working memory paradigm, Glahn *et al*. report clear support for hypofrontality, but also consistently increased activation of the anterior cingulate cortex and frontal pole in patients [67]. Consistent with this, aberrant frontotemporal functional connectivity patterns, with dorsal prefrontal/anterior cingulate hypoactivity were described in working memory tasks in patients compared to healthy controls [96]. Interestingly, an increase in functional connectivity between the DLPFC and hippocampus was found in patients [97]. Taken together, these findings could be suggestive of a disruption in frontal-based top-down cognitive control function and resulting compensatory response to support alternative strategies in task performance in patients with schizophrenia. In a meta-analysis of functional imaging studies of executive function in schizophrenia that also included a subset of studies using working memory tasks, Minzenberg *et al*. report widespread abnormalities of activation patterns, including deficits in the middle frontal gyrus, anterior cingulate cortex and thalamus in patients with schizophrenia compared to healthy controls. To maintain performance, patients may increase other processes including mnemonic, attentional and performance monitoring functions that manifest as relative hyperactivation in aforementioned regions; *i.e.*, less focused cortical activity [98].

Given the heritability of working memory deficits, several studies investigated the association between processing efficiency and catechol-O-methyltransferase (COMT), a known schizophrenia risk gene, during a working memory task. The authors did find the Val allele to be associated with a reduced level of performance and reduced efficiency of the physiological response in the dorsolateral prefrontal cortex, a finding that presumably is mediated by reducing signal to noise through compromising postsynaptic impact of evoked dopamine response [93,99,100,101,102,103]. When dopamine D1 receptor availability in the DLPFC was assessed in unmedicated patients, a relationship between increased receptor availability and worse working memory performance was identified, further substantiating the role of dopamine abnormalities in working memory deficits [104]. 

Several studies investigated effects of antipsychotic medications, which act by blocking dopamine receptors, on brain activation patterns in patients with schizophrenia. In a longitudinal study, Wolf *et al*. do report enhanced bilateral frontotemporal function after 7–8 weeks of multimodal antipsychotic treatment that was associated with improved accuracy in a verbal working memory task and improvement of psychotic symptoms [105]. Schlagenauf *et al*. report activation deficits during a working memory task in the DLPFC, but did not find significant changes in activation after switching patients from a typical antipsychotic to olanzapine. Surprisingly, they do report a normalization of frontal lobe activity when switching to aripiprazole, a partial dopamine agonist [106,107]. In another group of first-episode, medication naive patients, the baseline activation deficits in the DLPFC were unchanged after ten weeks of treatment with different second generation antipsychotics, an effect that was driven by medication non-responders. Patients who did respond to medications had DLPFC activation levels that were much more similar to that of healthy controls [108,109]. Other studies report improvement of baseline activation deficit in the DLPFC after a twelve week course of quetiapine and after 6–8 weeks of risperidone or olanzapine [81,110]. Consistent with this, Honey *et al*. did report improvement of functional activation by a working memory task in the right DLPFC within six weeks of switching from typical antipsychotics to risperidone and hypothesized that this finding may be due to reduced dopamine D2 receptor antagonism in the nigrostriatal system, which might enhance frontal cortical activity by reducing inhibitory outputs [111]. Contrastingly, regional cerebral blood flow abnormalities in the anterior cingulate cortex were corrected in patients treated with clozapine, but not haloperidol, suggesting differential mechanisms in mediating neural activation between these drugs [112]. 

### 2.2. Episodic Memory Deficits

A substantially smaller number of functional neuroimaging studies have been conducted with the goal to elucidate neural correlates of episodic memory deficits in schizophrenia. In the prefrontal cortex, activation deficits have most commonly been reported [73,113,114,115,116], but also increased activation, especially in the frontal pole, has been reported [113,116]. In addition to prefrontal abnormalities, some studies have also reported reduced activation of the MTL [73,115], with others reporting no change [87], or increased activation in this area [114,116]. 

Given that abnormalities appear to involve both the prefrontal cortex and MTL, a disruption in the frontotemporal network has been proposed [22]. In 14 patients with schizophrenia, Ragland *et al*. found evidence of this, reporting prefrontal activation deficits and parahippocampal over activation during encoding [114]. Evidence of reduced left inferior frontal activation during encoding and reduced anterior cingulate cortex and temporal lobe activation, but no reduction of hippocampal activation was later reported in a group of stable, medicated patients [117]. Similar to this, Hofer *et al*. report an activation deficit in the DLPFC and anterior cingulate cortex in encoding and impairments in DLPFC activation during retrieval. When encoding and recognition were contrasted, attenuated frontotemporal activation in patients with schizophrenia was apparent, despite intact behavioral performance [118]. Later, they also reported activation failure in the frontal, posterior cingulate and retrosplenial regions during encoding and reduced activation in the DLPFC and paralimbic regions during retrieval in acutely psychotic patients [119]. When patients were given organizational strategies through levels-of processing paradigms, patients had similar behavioral effects compared to healthy controls in a word encode and retrieve task that was accompanied by increased ventrolateral prefrontal activation during encoding in both groups. Overactivation in the thalamus, hippocampus and lingual cortex were present during encoding and overactivation of the left frontal pole with decreased activation of the right prefrontal cortex during retrieval were observed in patients with schizophrenia [116]. Hippocampal activity during deep encoding along with reduced anterior cingulate cortex and dorsomedial prefrontal cortex activation led to the hypothesis that an activation deficit in the anterior cingulate could result in insufficient top-down modulation of attention, resulting in impaired encoding performance [120]. Given the association between increased hippocampal activity, positive symptoms, and impaired memory performance, it is conceivable that increased neural activity is necessary to compensate for dysfunctional mesolimbic circuitry that results in a hyperdopaminergic state [121,122,123]. Both experimental and computational evidence suggests that excitation of the hippocampus can lead to excitation of dopaminergic neurons in the ventral tegmental area and, in turn, leads to increased release of dopamine in the hippocampus, possibly resulting in positive symptoms and memory impairment [121,124,125]. 

Meta-analytic evidence of episodic memory studies initially suggested reduced right hippocampal activation along with activation deficits of the left inferior frontal cortex and medial prefrontal cortex during encoding in patients with schizophrenia and a deactivation of the hippocampus, but hyperactivation of the parahippocampal gyrus with activation deficits of the anterior cingulate cortex, the inferior frontal cortex, middle frontal cortex and medial prefrontal cortex during retrieval [126]. However, a later meta-analysis investigating neuroimaging correlates of episodic memory deficits in schizophrenia did report less prefrontal activation in the frontal pole, DLPFC and ventrolateral prefrontal cortices during encoding and less DLPFC and ventrolateral prefrontal cortex during retrieval, but did not confirm reduced hippocampal or surrounding medial temporal lobe activation in patients during encoding or retrieval. They did find a relative increase in activation in the parahippocampal gyrus during encoding and retrieval, which may reflect a compensatory mechanism, with overall greater distribution in activation abnormalities in retrieval than encoding [127]. Discrepancies in findings of these meta-analyses may be associated with the difference in study inclusion criteria; with the latter excluding region-of-interest studies that the former had included. 

Genetic contributions to functional abnormalities with episodic memory tasks have been confirmed in a study of patients with schizophrenia and healthy siblings who demonstrated similar patterns of reduced hippocampal-parahippocampal activation with a task, suggesting a susceptibility-related phenotype [128]. In a similar approach, using a visual task, incremental increase in BOLD responses in schizophrenia compared with first-degree relatives and healthy controls in prefrontal regions, thalamus and insula were reported during retrieval, but no activation differences were noted during encoding, again highlighting a possible genetic component [129]. When directly testing effects of neuregulin, neurogranin and dystrobrevin-binding protein 1, all genes previously identified as increasing risk for schizophrenia have been demonstrated to modulate brain activation during episodic memory processing in healthy controls [130,131,132]. However, these links remain to be confirmed in patients with schizophrenia. An association that has been suggested to be relevant is the schizophrenia risk gene, COMT, that also been implicated in BOLD signal activation during working memory tasks. A genotype by diagnosis interaction has been demonstrated in investigating parahippocampal activation during encoding in patients with schizophrenia and healthy controls [133]. This finding again implies an alteration in dopamine signaling that could be related to observed abnormalities in brain activation patterns. 

Given this and the above discussed, evidence, albeit, not undisputed, of antipsychotic medications possibly affecting brain activation patterns in working memory, it is conceivable that these medications may also have modulating effects on activation during episodic memory tasks. While fMRI studies have enrolled subjects who were medicated [114,115,116,117,118] and unmedicated [119], there is a paucity of longitudinal investigations with the goal to elucidate changes of activation patterns induced by antipsychotic medications. 

### 2.3. Shared Abnormalities in Working Memory and Episodic Memory

Much of the work investigating memory deficits in schizophrenia has conceptualized working memory and episodic memory as two independent constructs and studied them individually. Given the shared neural substrates of both these memory systems in healthy controls and the evidence that prefrontal cortical abnormalities contribute to both working memory and episodic memory deficits, Barch *et al*. chose to test the hypothesis that abnormal prefrontal cortex activation contribute to deficits in both memory systems. Indeed, the authors found impaired activation in the right DLPFC in patients with schizophrenia in both tasks. Interestingly, all functional abnormalities observed in the working memory task were also present in the episodic memory task [73]. Congruent with this, working memory deficits in patients with schizophrenia were found to account for deficits in long-term recall in a behavioral study [134], but others report that these are differential core deficits in schizophrenia [135]. In a study investigating the relationship between neural processing related to working memory and long-term memory, Ragland *et al*. found that the DLPFC was activated during a working memory task, but that activation was less focal in patients compared to controls and that this activation did not translate to higher success in long-term memory processes. The authors further report that patients showed disproportional impairment in recognizing familiarity for items compared to general long-term memory deficits, emphasizing the processing of relational information in working memory [94]. It is conceivable that abnormal prefrontal cortex and MTL activation patterns and aberrant functional connectivity between these regions could be related to DLPFC dysfunction with subsequent compensatory mechanisms that result in difficulties both working memory and long-term memory deficits [136,137]. However, more investigations need to be conducted to clarify if both memory systems share a common neural substrate. 

## 3. Conclusions

Cognitive dysfunction is a critical and enduring feature of schizophrenia. Advances in neuroimaging methodology allow the study of memory processes *in vivo* and have made a tremendous contribution to our understanding of the functional alterations in the brain in schizophrenia. 

Numerous studies have been conducted to investigate neural correlates of two major memory systems affected in schizophrenia; working memory and episodic memory. Neural substrates of working memory deficits confirmed abnormalities in prefrontal activation patterns; most widely replicated in the DLPFC. Studies addressing the dynamic range of activation patterns in context of memory load suggest a non-linear, inverted U-shaped physiological response. In episodic memory, a disruption in frontotemporal connectivity is apparent. Interestingly, it seems that some of the activation abnormalities are shared in both memory systems, suggesting common neurophysiological underpinnings of working memory and episodic memory deficits in patients with schizophrenia. 

Emerging data suggests that dynamic changes occur in functional networks in response to underlying abnormalities in schizophrenia, but that these resulting networks are dysfunctional [28,92]. Compensatory mechanisms in schizophrenia may result in a limited improvement of performance through reweighting disturbed neural networks, but this circuitry is likely to be unstable and may fail to adapt to increasing cognitive demands [92]. 

Understanding the underlying neurobiology of memory deficits in schizophrenia remains a challenge, because functional magnetic resonance imaging studies are far from universal. Whether this is related to disease heterogeneity, differential response to pharmacologic treatment, effects of performance differences between groups, experimental design, image acquisition parameters or other factors yet to be elucidated is unclear. Clearly, brain regions are differentially affected in schizophrenia, but we lack an overarching mechanistic model of memory deficits that is inclusive of different memory systems, the relationship between memory systems and the modulatory capacity or non-memory-related neural networks [48]. It is critical to not only advance the understanding on the functional architecture of working memory dysfunction, but also to investigate the mechanisms that allow manipulation of related networks, potentially improving the treatment of schizophrenia. 
